# Cutaneous manifestation of leukaemia on the penis—the possible implications

**DOI:** 10.1093/jscr/rjaa549

**Published:** 2020-12-31

**Authors:** Angela Ng, Murat Gurun

**Affiliations:** Urology Department, University Ayr Hospital, Ayr KA6 6DX, UK; Urology Department, University Ayr Hospital, Ayr KA6 6DX, UK

**Keywords:** leukaemia, cutaneous manifestation, penis

## Abstract

Skin lesions are common in the patients with chronic lymphoid leukaemia (CLL); however, leukaemia cutis is a cutaneous manifestation secondary to any form of leukaemia and generally an uncommon phenomenon. They typically present on the face and neck as well as exposed areas. Our case looks at a 78-year-old gentleman with known CLL who presented with an asymptomatic raised lesion in his inner prepuce. The lesion was excised and his immunohistology staining confirms expression of CD5, CD20 and CD23 that is consistent with cutaneous manifestation of CLL. This case highlights the importance of taking leukaemia cutis into consideration in patients with known CLL with unusual features.

## CASE REPORT

A 78-year-old gentleman was referred to a Urology clinic presenting with a 3-month history of a 1-cm raised lesions appearing in the left side of his inner prepuce. On examination the lesion was described as pale, elliptical with no surrounding erythema, ulceration or signs of infection. There were no associated trauma symptoms, nor was any change in size over the course of the 3 months.

He has a background of Rai stage 0 chronic lymphoid leukaemia (CLL), diagnosed incidentally in 2006. He has been asymptomatic and never been commenced on treatment. Since his diagnosis has been on active monitoring every 6 months. Other than his CLL, he is otherwise fit man who does not smoke or drink any alcohol. His most recent blood results are shown in Table 1.

**Table 1:** Blood results at time of clinical review and post excision

Blood results prior to excision	Blood results post excision
Hb 134 WCC 35.4 Platelets 168 Lymphocytes 29.9	Hb 134 WCC 42.4 Platelets 167 Lymphocytes 42.4

Two months after clinic review, he had an excision of the cyst under local anaesthesia. The biopsies were analysed locally initially and then reconfirmed in a tertiary hospital. The pathology report showed dense diffuse lymphoid infiltrate that fills the papillary dermis and it also extends throughout the reticular dermis where it assumes a nodular perivascular and peri-adnexal distribution. Most of the infiltrate comprises uniform small lymphocytes and there were no sheet of blasts seen. In places these surround ill-defined pale staining aggregates of large cell consistent with proliferation centres. Occasional reactive lymphoid follicles and focal aggregate of plasma cells are also noted in the deeper aspect of the infiltrate. Most of the lymphocytes forming the infiltrate are of B-lineage with the following phenotypes:

- Positive: CD5, CD20, CD23, BCL2 and LEF1;
- Negative: CD3, CD10, BCL6 and Cyclin D1;
- Ki67 fraction: < 10%.

The features found on pathology were consistent in cutaneous infiltration by CLL.

The patient subsequently had an updated body CT scan and bloods. His blood results are shown in Table 1. His CT scan showed no significant axillary or mediastinal lymphadenopathy, with no evidence of progression. The patient continues to be under active surveillance by the Haematology team. There were no post-operative complications from the excision and was therefore discharged from Urology.

## DISCUSSION

CLL is the most common form of leukaemia in the western world showing an incidence of ~4.2 per 100 000 population [1]. Approximately 75% of cases are newly diagnosed in patients over 50 years with a peak age of 71 years.

Skin lesions are generally common in patients with CLL; however, this is on the background of trauma such as infection or new cutaneous malignancies such as squamous cell carcinoma. The term leukaemia cutis (LC) refers to cutaneous infiltration of leukaemia of any kind and is generally an uncommon presentation. It has been reported that cutaneous involvement can be seen in up to 15% of patients with known myeloid disorders and between 4 and 20% of patients with CLL [2, 3].

The dermatological features of LC are variable that make clinic diagnosis challenging. A case report of a patient with CLL presented with raised flesh-coloured nodules [3] that is not unsimilar in our patient. Locations are mainly found in the face, neck and exposed areas. There has been a case series of six patients with confirmed B-cell CLL where there has been leukaemia cutis in areas typical of Borrelia burgdorferi-induced pseudolymphomas, mainly the earlobes, nipples and scrotum; however, all these patients were found positive for Lyme disease [4]. In our patient’s case there was no history of Lyme disease.

There is little in the literature looking at CLL cutaneous lesions involving the penis—there are only four published cases where there have been confirmed CLL in the penis and where one of which also involves the prostate [5–8]. All four cases describe the lesion as ulcerated or an erosive dermatitic eruption. One case described the lesion spreading to the groin as well as the shaft of the penis whereas the remaining three were solitary lesions located in the shaft, foreskin or the glans. This certainly reflects on the variability of clinical features and the importance of immunohistological confirmation.

Our patient presented with the typical histological features of LC in CLL. This included patchy peri-adnexal and perivascular involvement that is modular and diffuses in nature. The diagnosis of CLL includes having positive immunophenotypes of lymphocytes (CD19, CD20, CD23 and CD5) and this was proven in the patient’s tissue biopsy.

Given the rarity of this manifestation, the current guidelines do not consider the LC as a drive for treatment. The indication for management is recommended by the NCI-sponsored Working Group [1] shown in Table 2.

**Table 2:** Indications for management of LC-NCI guidelines

The presence of B symptoms such as fever, sweats and weight loss Progressive enlargement of lymph nodes or hepatosplenomegaly Obstructive adenopathy Development of or worsening of thrombocytopenia and anaemia Immune haemolysis or thrombocytopenia not responsive to corticosteroid Rapid lymphocyte time

Treatments include reducing symptoms by the irradiating the enlarged spleen and lymph nodes. Alkylating agents, monoclonal antibodies and intravenous immunoglobulins are other possible therapies.

It has been well documented that LC reflects a poorer prognosis in those with acute myeloid leukaemia as this correlates with additional extramedullary involvement, however, the prognostic significance of LC in CLL is not entirely clear. A retrospective study of 42 patients by Cerroni *et al*. showed the 5-year survival rate was 66% and the prognosis significantly worsens in patients with Richter syndrome that is the transformation of CLL into mainly diffuse large B-cell lymphoma [9]. Yamazaki *et al.* revealed in their literature review that dermatological features of cutaneous Richter syndrome are non-specific and can be asymptomatic in patients with CLL [10]. Cutaneous Richter syndrome is generally a rare phenomenon, <20 cases have been reported in the literature, but it is associated with a grave outcome, with a mean survival of 8 months despite receiving aggressive chemotherapy.

LC is generally an uncommon presentation in patients with CLL and there is little in the literature to suggest any prognostic significance. Given the variability of the cutaneous features, immunohistology confirmation is strongly advised to not only exclude common causes of skin lesions such as infection but also to rule out phenomenon such as Richter syndrome that implies a graver prognosis and more aggressive treatment.

## CONFLICT OF INTEREST STATEMENT

None declared.
